# Pure retroperitoneal laparoscopic radical nephrectomy and thrombectomy with delayed occlusion of the proximal inferior vena cava (DOPI) technique for renal tumor with level II-III venous tumor thrombus

**DOI:** 10.1186/s12885-021-08392-5

**Published:** 2021-05-27

**Authors:** Zhuo Liu, Peng Hong, Guodong Zhu, Li Zhang, Xun Zhao, Shiying Tang, Feilong Yang, Xiaojun Tian, Guoliang Wang, Shudong Zhang, Hongxian Zhang, Yi Huang, Chunxia Liu, Cheng Liu, Lulin Ma

**Affiliations:** 1 Department of Urology, Peking University Third Hospital, 49 North Garden Road, Haidian District, Beijing, 100191 China; 2 Department of Ultrasound Diagnosis, Peking University Third Hospital, Beijing, China

**Keywords:** Inferior vena cava, Laparoscopic surgery, Renal tumor, Retroperitoneal approach, Tumor thrombus

## Abstract

**Purpose:**

To explore the safety and effectiveness of delayed occlusion of the proximal inferior vena cava (DOPI) technique in retroperitoneal laparoscopic radical nephrectomy (LRN) and thrombectomy for renal tumor with level II-III venous tumor thrombus (VTT).

**Materials and methods:**

From August 2016 to October 2018, a total of 145 patients with renal tumor and VTT were admitted to our centre. Seventy-five patients underwent laparoscopic surgery, and 70 patients underwent open surgery. Among these patients, 17 patients underwent retroperitoneal LRN and thrombectomy with the DOPI technique. Clinical data were collected retrospectively, and a descriptive statistical analysis was conducted.

**Results:**

All the patients successfully underwent retroperitoneal laparoscopic surgery. The mean operation time was 345.9 ± 182.9 min, the mean estimated blood loss was 466.7 ± 245.5 ml. Postoperative complications occurred in three patients, including two patients of Clavien grading system level IVa and one patient of level II. There were no complications related to carbon dioxide pneumoperitoneum, such as gas embolism, acidosis, and subcutaneous emphysema. During 21 months of median follow-up time, no local recurrence was found, and distant metastasis occurred in four patients. Cancer-specific death occurred in two patients.

**Conclusions:**

The DOPI technique is safe and feasible in the treatment of renal tumor and level II-III VTT. With the DOPI technique, the procedures of dissociating and exposing proximal inferior vena cava are simplified.

## Introduction

Renal tumors have the tendency to extend into the venous system. According to the Mayo classification, the tumor thrombus is classified as level 0 to IV [1]. In 1972 Skinner et al. reported the first radical nephrectomy with thrombectomy [2]. After that, the first case of laparoscopic radical nephrectomy (LRN) and thrombectomy for renal cell carcinoma (RCC) and renal vein tumor thrombus was reported in 1996 [3]. With the improvements in minimally invasive techniques, robotic-assisted laparoscopic radical nephrectomy (RLRN) and thrombectomy has been used to treat renal tumor and venous tumor thrombus (VTT). Presently, the feasibility and safety of LRN and thrombectomy have been confirmed by preliminary outcomes [4–6].

For the surgical treatment of renal tumor with level II-III VTT, dissociation of liver and occlusion of the first port and suprahepatic inferior vena cava (IVC) are routine steps. In this article, we introduce our pioneering delayed occlusion of the proximal inferior vena cava (DOPI) technique and its application in retroperitoneal LRN and thrombectomy for renal tumor and level II-III VTT.

## Materials and methods

### Patients

From August 2016 to October 2018, a total of 145 patients with renal tumor and VTT were admitted to our centre. Seventy-five patients underwent laparoscopic surgery, and 70 patients underwent open surgery. Among these patients, 17 underwent retroperitoneal LRN and thrombectomy with the DOPI technique. All the DOPI surgery was performed by an experienced surgeon, who specialized in complicated LRN and thrombectomy. The clinical data of the 17 patients, 9 men and 8 women, were collected retrospectively. All 17 patients had right renal tumor. According to Mayo classification [1], 13 patients were level II and 4 were level III. The present study was approved by the Institutional Ethics Committee of Peking University Third Hospital. Informed consent was obtained from all individual participants included in the study.

The inclusion criteria for the DOPI technique were as follows: VTT was level II-III; the retrohepatic and subhepatic segments of the IVC wall and hepatic vein wall were not invaded by tumor according to preoperative imaging examination; and no bland thrombus in the distal end of the IVC.

**Fig. 1:**
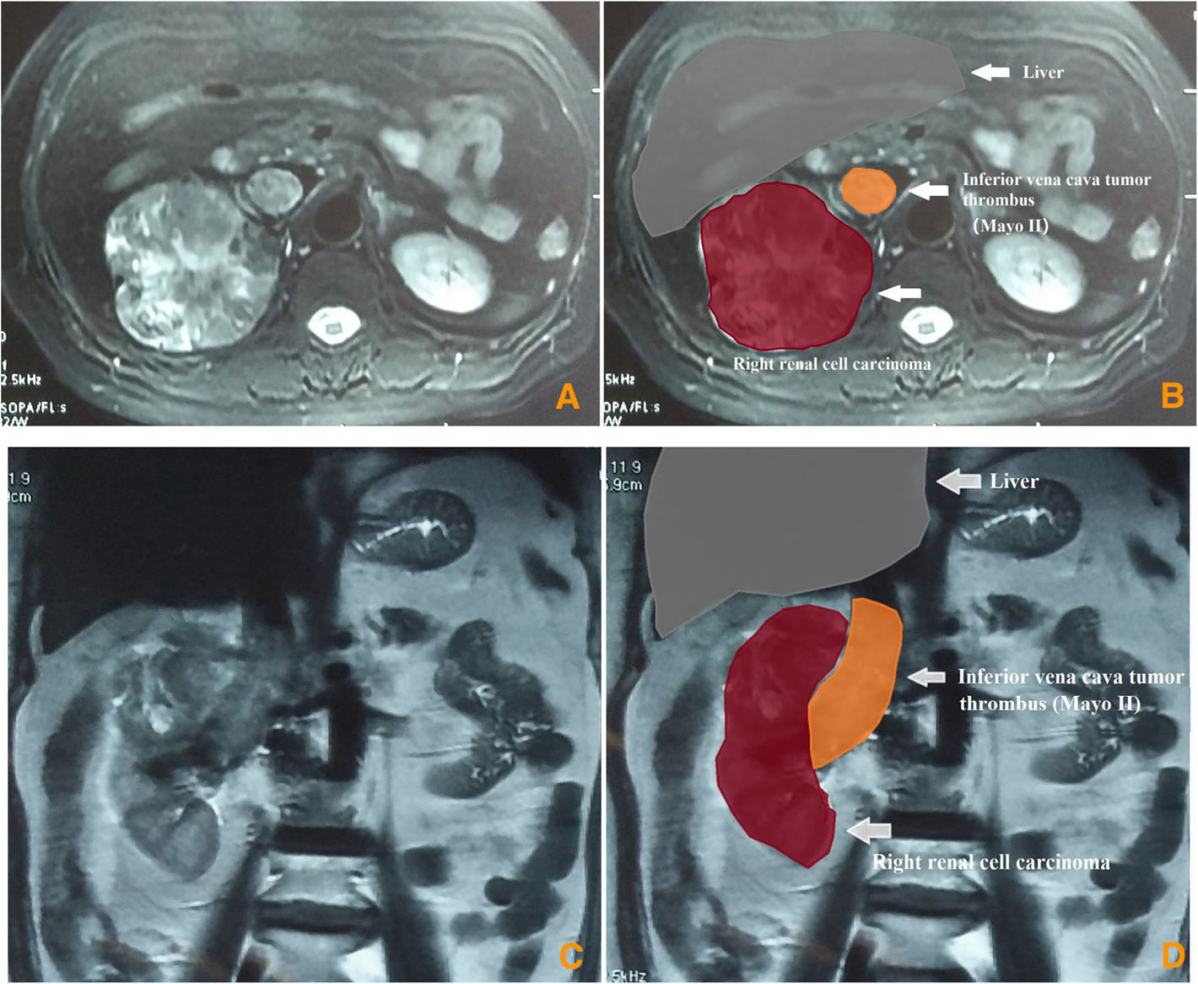
Renal tumor and inferior vena cava (IVC) tumor thrombus on the magnetic resonance imaging (MRI)

Before surgery, chest and abdominal computed tomography (CT) were performed. Enhanced magnetic resonance imaging (MRI) was performed to measure the length of VTT and assess tumor invasion of the IVC wall and hepatic vein wall (Fig. 1). Echocardiography was used to examine the heart function and assess the existence of atrial tumor thrombus. For patients with clinical symptoms, brain MRI and bone scan can be performed to assess brain metastases and bone metastases.

The follow-up, which included blood tests, chest radiography, and urinary enhanced CT, was performed every 6 months after surgery. All methods were carried out in accordance with relevant guidelines.

### Surgical technique

**Fig. 2:**
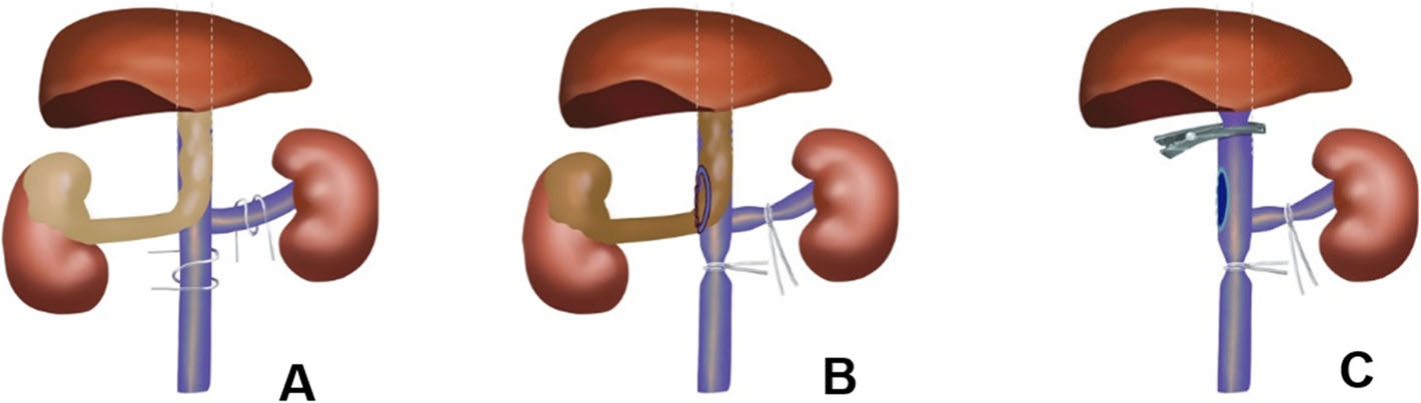
The delayed occlusion of the proximal inferior vena cava (DOPI) technique: (**A**). The distal end of the inferior vena cava (IVC) and the left renal vein were fully exposed, and blocking bands were placed 2 cm away from tumor thrombus and twined round vessels two circles; (**B**). The IVC was incised longitudinally at the entrance of the right renal vein into the IVC; (C). A bulldog clamp was put at the proximal end of the IVC

**Fig. 3:**
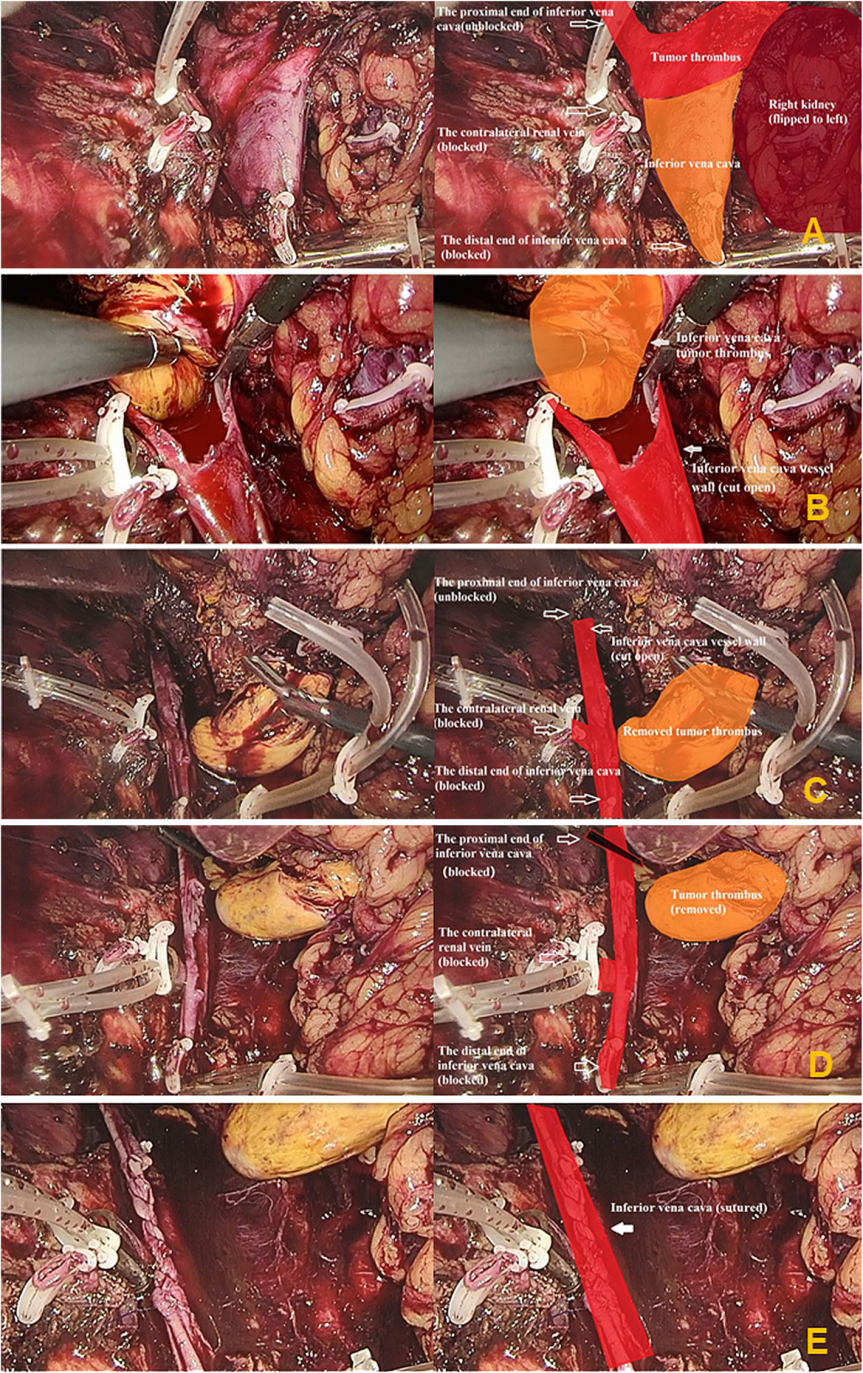
The key steps of the surgical procedure: (**A**). The distal end of the inferior vena cava (IVC) (under the renal vein) was fully dissociated, and a blocking band was placed. The left renal vein was fully dissociated approximately 2.5–3 cm in length for blocking. The proximal end of the IVC was exposed appropriately; (**B**). Blood filled into the IVC but did not flow out of the IVC; (**C**). The right renal tumor and tumor thrombus were removed completely; (**D**). A bulldog clamp was put at the proximal end of the IVC; (**E**). The incision of the IVC was sutured continuously with 4–0 Prolene vascular suture

The patient was placed in a left lateral position with the lumbar bridge elevated. A 2-cm longitudinal incision was made at the twelfth costal margin near the lateral edge of psoas. The fingers were used to separate and expand the retroperitoneum through the incision. The balloon was implanted and filled with 400 to 600 ml of air to dilate the retroperitoneum for 5 mins. A 13-mm trocar was inserted into the incision. Carbon dioxide pneumoperitoneum was established, and the pneumoperitoneum pressure was maintained at 12 mmHg. An 11-mm trocar was inserted above the iliac crest in the midaxillary line. A 5-mm trocar was inserted under the twelfth costal margin in the anterior axillary line. If necessary, a 5-mm trocar could be inserted approximately 5 cm inside the anterior superior iliac spine to assist the surgery. After carefully dissociating, the right hilar vessels and the lower pole of right kidney were exposed. The right renal artery was clamped and cut off. The ureter was dissociated approximately 7 to 8 cm in length from the lower pole of kidney and was clamped and cut off. The peritoneum in the right paracolic sulci was incised with an ultrasound scalpel, and then the right kidney was put into the peritoneal cavity through the incision to enlarge the space of the retroperitoneum. The distal end of the IVC (under the renal vein) and the left renal vein were fully exposed, and blocking bands were placed 2 cm away from tumor thrombus and twined round vessels two circles (Fig. 2a). The proximal end of the IVC was exposed appropriately (Fig. 3a). Short hepatic veins and lumbar veins were electrocoagulated to tawniness using bipolar electrocoagulation at the proximal and distal end of veins, and then were severed in the middle using ultrasonic scalpel with low gear. The distal end of the IVC and the left renal vein were blocked in sequence. The pneumoperitoneum pressure was increased to 15 to 25 mmHg. Then, the IVC was incised longitudinally with scissors at the entrance of the right renal vein into the IVC (Fig. 2b). During this procedure, the pneumoperitoneum pressure was adjusted to make sure that blood filled into the IVC but did not flow out of the IVC (Fig. 3b). The IVC wall that is invaded by the tumor thrombus was excised. The right kidney and VTT were removed completely (Fig. 3c). Then, a bulldog clamp was put at the proximal end of the IVC (Fig. 2c) (Fig. 3d). The pneumoperitoneum pressure was reduced to 12 mmHg. The lumen of the IVC was flushed with heparin saline, and the incision was sutured continuously with 4–0 Prolene vascular suture (Fig. 3e). The bulldog clamp on the proximal end of the IVC was loosened. The blocking bands on the left renal vein and distal end of the IVC were loosened in turn. Then, the pneumoperitoneum pressure was reduced to 5 mmHg, and we checked the tightness of incision. For bleeding site, medical gauze was used to press it, and then, the bleeding site was sutured with 4–0 Prolene vascular suture. After this, the drain tube was placed. A longitudinal incision along the muscle fibres was made in the waist, and the specimen was removed through the longitudinal incision. After this, the incision was closed.

## Results

### Patients characteristics

Detailed patient characteristics are shown in Table 1. The mean age of the 17 patients was 52.2 ± 26.9 yrs., and the mean body mass index (BMI) was 22.8 ± 4.4 kg/m^2^. The mean diameter of renal tumor was 8.1 ± 3.4 cm. The American Society of Anesthesiologists (ASA) score was I in 2 (11.8%) patients and II in 15 (88.2%) patients. In terms of clinical manifestations, there were three (17.6%) patients without clinical symptoms, eight (47.1%) patients with local symptoms, one (5.9%) patient with systemic symptoms, and five (29.4%) patients with both local and systemic symptoms. Before surgery, there were 7 (41.2%) patients with lymph node involvement, and 3 (17.6%) patients with distant metastasis.

**Table 1:** Clinical characteristics of patients undergoing pure retroperitoneal laparoscopic radical nephrectomy and thrombectomy with delayed occlusion of the proximal inferior vena cava (DOPI) technique

Variables	Value
Patients, n	17
Age, yr, mean ± SD (range)	52.2 ± 26.9 (18–79)
BMI, kg/m^2^, mean ± SD (range)	22.8 ± 4.4 (15.2–32.4)
Sex, n	
Male	9
Female	8
Side, n	
Right	17
Clinical symptoms, n (%)	
No clinical symptoms	3
Local symptoms	8
Systemic symptoms	1
Both	5
ASA score, n (%)	
1	2
2	15
Tumor diameter, cm, mean ± SD (range)	8.1 ± 3.4 (3.5–11)
Lymph node involvement, n	7
Distant metastasis, n	3
Mayo classification, n	
II	13
III	4

*SD* standard deviation; *BMI* body mass index; *ASA* American Society of Anesthesiologists

### Outcomes

All the 17 patients successfully underwent pure retroperitoneal laparoscopic surgery with the DOPI technique, and no patient was converted to open surgery. Detailed surgical and oncological outcomes are stated in Table 2. No patient developed gas embolism or carbon dioxide poisoning. The mean operative time was 345.9 ± 182.9 mins, and the mean estimated blood loss was 466.7 ± 245.5 ml. Eight patients had intraoperative transfusion of red blood cells, and the mean transfusion volume was 1200 ± 1054.2 ml. Four patients had intraoperative transfusion of plasma, and the mean transfusion volume was 475 ± 82.9 ml. Three (17.6%) patients underwent IVC resection because of the invasion of tumor thrombus. In terms of pathological type, there were 12 (70.6%) patients with clear cell RCC, 1 (5.9%) patient with Ewing sarcoma, 1 (5.9%) patient with papillary RCC, 1 (5.9%) patient with chromophobe RCC, 1 (5.9%) patient with urothelial carcinoma, and 1 (5.9%) patient with angiomyolipoma. Postoperative complications occurred in three (17.6%) patients, and the complications were graded according to the Clavien grading system [7]. One patient had acute cerebral infarction (grade IVa), one patient acute renal insufficiency (grade Iva) and one patient respiratory tract infection (grade II). There was no anticoagulant therapy in the postoperative period. The mean postoperative hospital stay was 9.3 ± 4.2 days. The preoperative mean serum creatinine was 88.5 ± 29.4 μmol/L, and the mean serum creatinine 1 week after surgery was 107.2 ± 64.9 μmol/L.

**Table 2:** Perioperative data and postoperative pathological data of patients undergoing pure retroperitoneal laparoscopic radical nephrectomy and thrombectomy with delayed occlusion of the proximal inferior vena cava (DOPI) technique

Variable	Value
IVC resection, n	3
Operative time, min, mean ± SD (range)	345.9 ± 182.9 (240–464)
Estimated blood loss, ml, mean ± SD (range)	466.7 ± 245.5 (200–1000)
Intraoperative transfusion of red blood cells, ml, mean ± SD (range) (*n* = 8)	1200.0 ± 1054.2 (400–3100)
Intraoperative transfusion of plasma, ml, mean ± SD (range) (*n* = 4)	475 ± 82.9 (400–600)
Postoperative hospital stay, day, ml, mean ± SD (range)	9.3 ± 4.2 (5–19)
Postoperative complications, n	3
Preoperative serum creatinine, μmol/L, mean ± SD (range)	88.5 ± 29.4 (48–117)
Serum creatinine 1 week after surgery, μmol/L, mean ± SD (range)	107.2 ± 64.9 (67–323)
Pathological type, n	
Clear cell RCC	12
Ewing sarcoma	1
Papillary RCC	1
Chromophobe RCC	1
Urothelial carcinoma	1
Angiomyolipoma	1
Follow-up time, mo, median (range)	21 (6–41)

*IVC* inferior vena cava; *SD* standard deviation; *RCC* renal cell carcinoma

The follow-up time of the 17 patients was 6 to 41 months, and the median follow-up time was 21 months. Two patients died of cancer within 1 year after surgery, and the follow-up time of remaining patients is more than 1 year. One patient was pathologically diagnosed with ewing sarcoma, and developed lung metastasis after surgery. She had no lymph node involvement and distant metastasis before surgery. The other one was pathologically diagnosed with chromophobe RCC, and developed bone metastasis after surgery. He had lung metastasis and no lymph node involvement before surgery. During follow-up, no local recurrence was found. Distant metastases were found in four patients after surgery and three patients were alive. They were pathologically diagnosed with clear cell RCC. They had lymph node involvement and no metastasis before surgery. After surgery, two patients developed lung metastasis, and one patient developed liver metastasis.

## Discussion

Surgical treatment for renal tumor with VTT is technically demanding and challenging. However, radical nephrectomy with thrombectomy could provide a good prognosis and is the main treatment option for renal tumor and VTT.

With the development of minimally invasive technique, laparoscopic surgery has gradually been used in the treatment of renal diseases, including complex renal tumor [8, 9]. It is reported that laparoscopic surgery could reach outcomes equal to open surgery [10]. Moreover, laparoscopic surgery has some advantages over open surgery, including smaller incision, less blood loss, less pain, shorter hospitalisation, and shorter recovery time. In 2006 Romero et al. first described pure LRN and thrombectomy for RCC with level II VTT [11]. Then, an increasing number of studies have established the efficacy and safety of LRN and thrombectomy for the treatment of RCC patients with level II-III VTT [12, 13]. In this study, we introduce our modification in the treatment of LRN and thrombectomy for renal tumor and level II-III VTT and present our preliminary results.

Both retroperitoneal and transperitoneal approaches have been reported to be used in LRN and thrombectomy [14–17]; thus, it is the preference of surgeon that decides the selection of surgical approach. In our centre, we performed LRN and thrombectomy using the retroperitoneal approach, because we have extensive experience in retroperitoneal laparoscopic surgery. The retroperitoneal approach could provide more direct and quicker access to the renal artery and lumbar veins [18]. The early occlusion of the renal artery could result in less intraoperative bleeding and lower risk of tumor embolism. The retroperitoneal approach does not disturb intraabdominal organs, and provides good exposure to the retroperitoneal vessels. Moreover, the retroperitoneal approach is not affected by the history of abdominal surgery and abdominal adhesion [17].

The conventional occlusion technique requires blocking the distal end of the IVC, the contralateral renal vein and the proximal end of the IVC before thrombectomy [5, 13, 19–23]. Compared with conventional LRN and thrombectomy for renal tumor with level II-III VTT, the essential difference of our pioneering technique is that the proximal end of the IVC is not clamped. During the surgery, the surgeon can elevate the pressure of the pneumoperitoneum to achieve the occlusion of the proximal end of the IVC. The pressure of the pneumoperitoneum is usually maintained at 15 to 25 mmHg. The lumen of the IVC maintains blood filling and not outflowing. If there is outflowing of blood, the pressure is elevated and often raised to 21 to 25 mmHg. During this procedure, the suction apparatus remains closed as much as possible to avoid interfering with the pressure of the pneumoperitoneum. After excision of the VVT, a bulldog clamp is put on the proximal end of the IVC to prevent bleeding. Not all patients with renal tumor and VVT can be treated using the DOPI technique. The DOPI technique applies only to patients whose retrohepatic and subhepatic segments of IVC wall are not invaded by tumor. We use CT and MRI to confirm the extent of VTT before surgery. Further, intraoperative ultrasound, typically used to assess the exact extent of VTT, is not necessary. With our DOPI technique, the dissociation of the liver is unnecessary. We do not need to disconnect the round, right and left triangular coronary and falciform ligaments. And the dissociation of the first port and suprahepatic IVC is unnecessary. Thus, the incidence of hepatic injury and bleeding volume decrease. The estimated blood loss in our study is less, and the mean estimated blood loss is 466.7 ± 245.5 ml. In addition, no tumor embolism or air embolism occurred in our patients. Compared with the outcomes of conventional occlusion technique (Table 3), the outcomes of DOPI technique were acceptable.

**Table 3:** The perioperative outcomes and oncological outcomes of conventional occlusion technique

Study	Surgical method	Surgical technique for TT	Case number (L/R)	OT (min), median (range)	EBL (ml), median (range)	Occluded time of IVC (min), median (range)	Complication Grade (n)	Mayo classification (n)	Pathology	Follow-up time (month), median (range)	Prognosis
Hoang AN et al. [5]. 2010	LS	CO with a Satinsky or similar clamp	0/6	240 (159–330)	550 (400–4250)	Na	IV:2	II:5 III:1	Na	15 (6–42)	One lung metastasis and died
Shao P et al. [19]. 2015	LS	CO with bulldog clamp (vessel loop)	0/6	155 (135–210)	275 (150–510)	16.5 (13–20)	I-II:2	II:6	NA	32.5 (16–52)	Disease free
Bratslavsky G et al. [20]. 2015	RALS	CO with vessel loop	0/1	366	1200	NA	No	III:1	Clear cell RCC	20	Disease free
Wang M et al. [13]. 2016	LS	CO with tourniquet loop	0/5	241 (180–300) (mean)	290 (50–1000) (mean)	NA	II:1	II:5	Clear cell RCC	11.5 (5–30) (mean)	Lung metastasis
Ramirez D et al. [21]. 2016	RALS	CO with vessel loop	0/1	353	150	39	NA	III:1	Clear cell RCC	NA	NA
Chopra S et al. [22]. 2017	RALS	CO with vessel loop	7/17	270 (180–480)	240 (100–7000)	24 (18–102)	II:2 III:2	II:13 III:11	RCC:23 Papillary type II RCC:1	16 (12–39)	11 recurrence
Wang B et al. [23]. 2018	RALS	CO with vessel loop	6/16	285 (191–390)	1350 (1000–2075)	28.5 (21–31.3)	I:3 II:5 IV:5	II:20 III:2	Clear cell RCC:16 Papillary RCC:2 XP11.2-related RCC:1 Carcinoma of the renal pelvis:1 Carcinosarcoma:1	16.8 (12–36).	One died, one recurrence, three progressed

*LS* laparoscopic surgery; *RALS* robot-assisted laparoscopic surgery; *TT* tumor thrombus; *L* left; *R* right; *OT* operative time; *EBL* estimated blood loss; CO conventional occlusion; *NA* not available; *RCC* renal cell carcinoma

Based on our initial experience, there are some important steps that should be taken into consideration in this procedure. First, after mobilisation of the kidney, we incise the peritoneum near the paracolic sulci and put the kidney into the peritoneal cavity through the incision. Thus, the space of the retroperitoneal cavity becomes larger, and the IVC and contralateral renal vein can be better exposed. A larger retroperitoneal cavity is conducive to the surgery. During this process, gentle operation is necessary, and unnecessary touch of IVC should be reduced to reduce the possibility of thrombus embolism. Second, short hepatic veins and lumbar veins are electrocoagulated to tawniness using bipolar electrocoagulation at the proximal and distal ends of veins before severed, which helps to avoiding bleeding caused by Hemolok clips that might fall off during surgery. For lumbar veins, they should be severed about 2 mm from the IVC with ultrasonic scalpel. If there is bleeding at the proximal end of veins, it was sutured with 4–0 Prolene vascular suture. Third, the vessel loop is not used to clamp the IVC and contralateral renal vein during surgery. We cut the smallest segment of the transfusion set, and use it to block the IVC and contralateral renal vein. The transfusion set is relatively harder than vessel loop, and it is easier to be manipulated. Fourth, our longitudinal waist incision could reduce damage to peripheral nerves and muscle fibres, and thus, decrease the incidence of incisional hernia and the degree of lower abdominal pain. In our study, no abdominal hernia occurred in the patients, and any discomfort in the lower abdomen was not serious.

We acknowledge some limitations in our study. First, the sample size of this study is small. A larger series of patients is required to verify the efficacy and safety of our DOPI technique. Second, our median follow-up time of 21 months is short, and we cannot evaluate the long-term oncologic outcomes. Third, the IVC occluded time is important in the LRN and thrombectomy, but our related data are absent. In a word, further studies with longer follow-up time and larger sample size are needed to assess this technique.

## Conclusions

The DOPI technique is safe and feasible in the treatment of LRN and thrombectomy for renal tumor and level II-III VTT. And, the preliminary outcome is satisfactory. With the DOPI technique, the procedures of dissociating and exposing the proximal end of the IVC is simplified, and the incidence of hepatic injury and bleeding volume may decrease. However, in complex cases, such as giant tumor, invasion of the circumambient organ and the invasive extent of IVC wall up to hepatic vein level, open surgery is essential.

## Data Availability

The datasets used and/or analysed during the current study are available from the corresponding author on reasonable request.
